# Enhancing control of multidrug-resistant plasmid and its host community with a prolonged thermophilic phase during composting

**DOI:** 10.3389/fmicb.2022.989085

**Published:** 2022-08-17

**Authors:** Lei Shen, Tianlei Qiu, Yajie Guo, Min Gao, Haoze Gao, Guozhu Zhao, Xuming Wang

**Affiliations:** ^1^National Engineering Research Center of Tree Breeding and Ecological Restoration, College of Biological Sciences and Technology, Beijing Forestry University, Beijing, China; ^2^Beijing Key Laboratory of Agricultural Genetic Resources and Biotechnology, Institute of Biotechnology, Beijing Academy of Agriculture and Forestry Sciences, Beijing, China; ^3^College of Life Sciences, Langfang Normal University, Langfang, China

**Keywords:** antibiotic resistance genes, multidrug-resistant plasmid, horizontal gene transfer, phylogenetic analysis, manure composting

## Abstract

The plasmid-mediated horizontal transfer of antibiotic resistance genes (ARGs) among bacteria facilitates the evolution and dissemination of antibiotic resistance. Broad-host-range plasmids can be transferred to different bacterial hosts in soil, plant rhizospheres, and wastewater treatment plants. Although composting is an effective way to convert organic waste into fertilizer and reduce some ARGs, few studies have focused on its effects on the spread of ARG-carrying plasmids and their bacterial host communities during composting. In this study, a fluorescently labeled *Pseudomonas putida* (*P. putida*) harboring a broad-host-range plasmid RP4 carrying three ARGs was inoculated into a raw material microcosm and composted with different durations of the thermophilic phase. The fate of the donor and RP4 in composting was investigated. The prolonged thermophilic composting removed 95.1% of *dsRed* and 98.0% of *gfp*, and it inhibited the rebound of *P. putida* and RP4 during the maturation phase. The spread potential of RP4 decreased from 10^−4^ to 10^−6^ transconjugants per recipient after composting. In addition, we sorted and analyzed the composition of RP4 recipient bacteria using fluorescence-activated cell sorting combined with 16S rRNA gene amplicon sequencing. The recipient bacteria of RP4 belonged to eight phyla, and Firmicutes, accounting for 75.3%–90.1%, was the dominant phylum in the transconjugants. The diversity and richness of the RP4 recipient community were significantly reduced by prolonged thermophilic periods. Overall, these findings provide new insights for assessing the contribution of composting in mitigating the dissemination of plasmid-mediated ARGs, and the prolonged thermophilic phase of composting can limit the transfer of multidrug-resistant plasmids.

## Introduction

Livestock manure is considered an important source of environmental antibiotic-resistant bacteria and antibiotic resistance genes (ARGs) because of the worldwide usage of antibiotics in intensive animal production (Vikesland et al., 2017; Qian et al., 2018). Manure aerobic composting is an effective technology for producing fertilizer and removing antibiotic residues; however, the fate of ARGs is complicated because of diverse abiotic and biotic factors during composting (Liu et al., 2021). Based on correlation analysis, researchers found that mobile genetic elements (MGEs) and potential bacterial hosts are two crucial biotic factors for the removal of ARGs by composting (Liao et al., 2019a; Cao et al., 2020; Qiu et al., 2021; Xie et al., 2022). In addition, horizontal gene transfer (HGT) has recently been observed as a key determinant of ARG proliferation in composting (Liao et al., 2019a; Zhou et al., 2021). Among HGT processes between bacteria, plasmid-mediated conjugation is the most prevalent mechanism of horizontal transfer of ARGs, especially broad-host plasmids, which can transfer ARGs between different phyla (Che et al., 2021). Manure from intensive animal farms is a reservoir of broad-host plasmids carrying multidrug-resistant genes, which greatly increases the probability of other bacteria acquiring multidrug resistance through HGT (Binh et al., 2008; Yang et al., 2017). Moreover, after the current manure composting process, some antibiotic-resistant plasmids in composts could still be captured (Le Devendec et al., 2016), and ARGs and plasmids in composts could also be transferred to the soil and plant endosphere (Guo et al., 2021; Sun et al., 2021; Xu et al., 2021). To prevent the spread of ARGs from animal manure, it is essential to evaluate the contribution of composting to mitigate the dissemination of plasmid-mediated ARGs.

To understand the fate of antibiotic-resistant plasmids during composting, it is necessary to decipher the relationship between ARG-carrying plasmids and their bacterial host communities. Network analysis based on the correlation between ARGs and bacterial communities showed that some ARGs increased with potential hosts after composting (Su et al., 2015; Sardar et al., 2021). Conjugation studies have shown that conventional composting fails to control the transfer risk of sulfonamide- or colistin-resistant plasmids in cow or broiler manure (Le Devendec et al., 2016; Lin et al., 2021). Metatranscriptomic analysis found that the microbial community was the key factor defining the transcriptional responses of ARGs during composting (Wang et al., 2017). These studies have shown that the shift in microbial hosts directly affects the fate of ARGs in composting. However, there is no direct evidence of plasmid-mediated ARG transfer and changes in conjugative plasmid receipts. Combining fluorescent reporter genes-based fluorescence-activated cell sorting (FACS) with 16S rRNA gene amplicon sequencing made it possible to directly track the dissemination of ARG-carrying plasmids among indigenous bacteria in the soil (Klümper et al., 2015, 2017; Fan et al., 2019), plant rhizosphere (Xu et al., 2021), and wastewater treatment plants (Li et al., 2018). However, few studies have focused on the spread of ARG-carrying plasmids and their bacterial host communities during composting.

The composting temperature intensity and duration of the thermophilic phase are important for the effective removal of ARGs (Liu et al., 2021). Hyperthermophilic composting and prolonged thermophilic phase could promote the removal efficiency of ARGs/MGEs and reduce the abundance of potential ARG bacterial host taxa based on correlation analysis (Qian et al., 2016; Liao et al., 2019b; Keenum et al., 2021). A culture-dependent conjugative study showed that high temperatures could help limit the spread of plasmid-carrying ARGs with *Escherichia coli* in chicken manure composting (Guan et al., 2007). Evaluating the community shifts in ARG-carrying plasmid receipts will provide direct evidence for the control of HGT in manure composting.

Therefore, this study reports the first sight of bacterial host community shifts toward the typical conjugative plasmid (RP4) and the first exploration of the effect of a prolonged thermophilic phase to prevent the spread of ARG-carrying plasmids during composting. We evaluated the impact of a prolonged thermophilic phase on the fate and persistence of donor and RP4 plasmids during composting. The abundance of donors and plasmids was tracked by digital droplet PCR (ddPCR), and the transconjugants were analyzed by FACS and Illumina sequencing. The primary aims of this study were (1) to investigate the effects of a prolonged thermophilic phase on the removal of RP4 plasmid and donor and (2) to determine the roles of the prolonged thermophilic phase in reducing the potential for ARGs spread through conjugation. These findings provide new insights into manure treatment strategies to mitigate the potential for HGT of ARGs.

## Materials and methods

### Donor strain and plasmid

*Pseudomonas putida* KT2442 harboring a broad-host-range IncP plasmid RP4 was used as the donor, which was gifted by Professor Barth F. Smets (Musovic et al., 2010). This bacterium was marked with *Discosoma* sp. red fluorescent protein (DsRed) gene and *lacI^q^* in the chromosome. Plasmid RP4 (carring the kanamycin, ampicillin, and tetracycline resistance genes) was tagged with *gfp* gene, encoding the green fluorescent protein (GFP), and its promoter was repressed by *lacI^q^*. Therefore, *P. putida* only exhibited red fluorescence due to *gfp* expression being repressed in the donor strain. Nevertheless, when the plasmid is transferred to a recipient, the *gfp* gene is derepressed owing to a lack of *lacI^q^*, and green fluorescence is expressed, which can be retrieved by FACS or detected by confocal microscopy (Sørensen et al., 2005).

*Pseudomonas putida* KT2442 was grown aerobically at 35°C and 180 rpm overnight in lysogeny broth supplemented with 100 μgml^−1^ ampicillin, 50 μgml^−1^ kanamycin, and 20 μgml^−1^ tetracycline. Bacterial cells were then harvested by centrifuging at 10,000× *g* for 10 min and washed three times by resuspending in sterile phosphate-buffered saline (PBS). The bacterial concentration was measured by counting the colony forming units (CFU).

### Composting system and raw material

The experiment was performed in a 50-L bench-scale cylindrical stainless steel composting reactor equipped with a computer control. Aeration was provided by an air pump connected to a pipe equipped at the bottom of the reactor. A channel for air exhaust was present at the top of the reactor. The aeration rates were controlled by a gas flowmeter. The reaction temperature was controlled *via* resistive heating, using a custom-made computer-controlled power supply. A schematic of the compost reactor is shown in Supplementary Figure S1.

The composting raw material consisted of chicken manure and corn straw (crushed to 0.5–1 cm), both obtained from a commercial poultry farm in Beijing, China. Chicken manure and corn straw were mixed in a 3.5:1 ratio (w/w), and the moisture content was adjusted to ~60% with sterile water. After thorough mixing, 28 kg of the mixed raw material was transferred into each composting reactor. Compost microcosms were prepared by thoroughly mixing the donor bacterial suspension with 2 kg of raw materials, providing a final donor strain density of 1.5 × 10^7^ CFUg^−1^ dry weight (DW). The prepared compost microcosms were packed into nylon mesh bags (40 cm long, 30 cm wide, mesh size: 2 mm) and placed in the center of the reactor. Control compost microcosms (CK) without donor inoculation were prepared and incubated at room temperature.

### Experiment design

To investigate the effects of temperature on composting, two treatments were set up: normal thermophilic composting (NT treatment) and continuous thermophilic composting (CT treatment). Each composting treatment consisted of three replicate reactors. The NT treatment was run naturally during the entire composting process, and it had a natural thermophilic phase; for the CT treatment, the reactor was controlled and maintained at approximately 55°C for an additional 15 days after the natural thermophilic phase, and then the composting mixture was poured out of the reactor and matured at room temperature for 13 days.

The temperature of the composting mixture was monitored and recorded daily using an automatic temperature sensor (Supplementary Figure S2). The aeration rate was maintained at 0.5 l/min/kg DW throughout the composting process (Wang et al., 2013). The compost piles and microcosms were manually turned three times per week.

NT treatment was performed for 27 days, and CT treatment was performed for 40 days. Compost microcosms were collected from nylon mesh bags in each reactor on days 0, 1, 4, 7, 11, 15, 19, 27, and 40 and divided into two subsamples: one was stored at 4°C for bacterial cell extraction and the other was stored at −80°C for subsequent DNA extraction.

### DNA extraction and droplet digital PCR

For compost samples, DNA was extracted according to Sun et al. (2019). The absolute abundances of *P. putida* and RP4 plasmid were performed by quantifying the *dsRed* and *gfp* genes, respectively. The *dsRed*, *gfp,* and 16S rRNA gene copies in samples were quantified by droplet digital PCR (ddPCR, QX200, Bio-Rad). The primer sequences and annealing temperatures for each tested gene are presented in Supplementary Table S1. Each ddPCR mixture consisted of 10 μl of 2 × QX200 ddPCR EvaGreen Supermix, 100 nM of each primer, and 1 μl of sample DNA. The final volume (20 μl) was prepared using nuclease-free water. The reaction mix was converted into droplets using the Bio-Rad QX200 automatic droplet generator and amplified in a Bio-Rad C1000 thermocycler under the following conditions (ramping rate 2.5°C/s): one cycle at 95°C for 5 min (denaturation), 40 cycles of 95°C for 30 and 60 s at the respective annealing temperatures (Supplementary Table S1), and one final cycle of 4°C for 5 min and 90°C for 5 min. Data were analyzed with the QuantaSoft Software version 1.7 (Bio-Rad).

The absolute abundances of donor *P. putida* and plasmid RP4 in the compost samples were transformed into lg(*C_t_/*C*_0_*), where *C_0_* and *C_t_* represent the initial abundances and abundances at time t (days), respectively (Fan et al., 2019). The “spread potential (SP)” was used to denote the dissemination of RP4 plasmid in the compost microbiota. SP refers to transconjugants/recipients, and is roughly calculated by the following formula:


$${\text{SP}}_{\text{ddPCR}}\text{ = }\left({\text{C}}_{gfp}-{\text{C}}_{dsRed}\right)/\left({\text{C}}_{\text{16SrRNA}}/4-{\text{C}}_{dsRed}\right)\text{.}$$

C*_gfp_* − C*_dsRed_* represents the number of transducers per gram of DW, assuming that each donor strain harbors a plasmid. According to the ribosomal RNA database, the average copy number of 16S rRNA genes per bacterium is estimated as 4.1 (Klappenbach et al., 2001). C_16SrRNA_/4 represents the total number of bacteria per gram DW.

The concentrations of the donor *P. putida* and plasmid RP4 in the compost samples were transformed using the logarithmic function before statistical analysis. The means and standard deviations were determined, and linear regression modeling was used to describe the decay of *P. putida* and plasmid RP4 during composting. Decimal reduction times (D-values; the time taken to reduce the concentration of *dsRed* and *gfp* by 1 log) for all composting treatments were calculated using the absolute estimated inverse values of the slope of the linear regression analyses.

### Extraction of bacteria from compost

Bacteria from the collected compost samples were harvested using Nycodenz density-gradient centrifugation (Musovic et al., 2010) and resuspended in PBS. Compost samples (1.0 g) were homogenized in 4 ml of tetrasodium pyrophosphate (TTSP, 50 mM, and Tween 80, 0.05%), vortexed and sonicated in an ice-cold water bath (Ultrasonic Cleaner KQ-500B, Kunshan, China) for 10 min. After 10 s of vortexing, the suspension was carefully transferred to Nycodenz (Nycomed Pharma, Oslo, Norway) solution with a density of 1.3 gml^−1^, followed by centrifugation (10,000× *g* for 10 min). The upper and middle phases containing a bacterial layer were collected and transferred to a new tube, diluted 1:5 (v/v) in PBS, and centrifuged (5,000× *g* for 10 min). The supernatant was discarded and the pellet resuspended in 1 ml of PBS, and filtered by sterile 20-μm-pore-size filters. After filtration, the suspensions were analyzed using flow cytometry.

### Flow cytometry sorting of transconjugants

The transconjugants with the obtained RP4 plasmid in the compost were sorted on an S3e Cell Sorter (Bio-Rad, Hercules, CA, United States). GFP fluorescence was excited with a 488 nm laser beam and checked in a band-pass filter 1 (525/30 nm), whereas the fluorescence of dsRed was excited at 561 nm and collected with filter 2 (586/25 nm). *E. coli* MG1655 and *E. coli* MG1655 (RP4::*gfp*) were used as the negative control for fluorescence and *gfp*-positive control, respectively, and *P. putida* KT2442 (RP4::*gfp*) was used as *gfp*-negative and *dsRed*-positive controls. The signal cells were gated based on forward scatter (FSC) and side scatter (SSC), filter 1, and filter 2. The cells with high green fluorescence and no red fluorescence were sorted and collected in a 5 ml sterile polystyrene round-bottom tube (Falcon, Corning, Wiesbaden, Germany) with 0.5 ml sterile PBS solution. The sort precision was set in the default purity mode (Fan et al., 2019).

### Bacterial cell lysis, 16S rRNA gene amplification, and sequencing

Sorted cells of each sample were initially collected in 5 ml sterile round-bottom polystyrene tubes (Falcon, Corning, Wiesbaden, Germany) with 0.5 ml of PBS solution, and then centrifuged at 10,000× *g* for 30 min in 1.5 ml tubes to collect the pellets (Klümper et al., 2015). The supernatant was carefully discarded, and the cell pellet was resuspended in 20 μl of lysis buffer (Platinum™ Direct PCR Universal Master Mix, Invitrogen, Thermo Fisher, United States) and 0.6 μl of proteinase K. The lysis mixtures were incubated at 25°C for ≥1 min, and the samples were placed in a pre-heated heat block and incubated at 98°C for 1 min. The DNA-containing cell lysate was centrifuged, and the lysate supernatant was used directly for subsequent PCR. The V3-V4 region of the bacterial 16S rRNA gene was amplified using universal primers (338F and 806R) and KAPA HiFi HotStart ReadyMix (KAPA Biosystems). The PCR thermocycler conditions were as follows: initial denaturation at 95°C for 3 min; 29 cycles at 95°C for 30 s, annealing at 55°C for 30 s, extension at 72°C for 45 s; with a final extension at 72°C for 10 min. Amplicons were purified by gel extraction using the AxyPrep DNA Gel Extraction Kit (Axygen) and quantified using QuantiFluor-ST (Promega, Madison, WI, United States). Purified amplicons were pooled in equimolar concentration and paired-end sequenced by the Illumina MiSeq PE 300 platform (Shanghai Majorbio Bio-Pharm Technology).

Raw fastq sequencing data were trimmed and quality-filtered using FastP version 0.20.0 (Chen et al., 2018) and merged with FLASH version 1.2.7 (Magoč and Salzberg, 2011). Operational taxonomic units (OTUs) were clustered with a 97% similarity cutoff using UPARSE version 7.1 (Edgar, 2013), while the chimeric sequences were identified and removed using UCHIME (Edgar et al., 2011). OTU was classified by the RDP Classifier 2.2^1^ against the Silva (SSU138) 16S rRNA database using a confidence threshold of 70% (Wang et al., 2007). The indices of alpha diversity including Sobs, Chao, Ace, Shannon, Simpson, and Good’s coverage were analyzed by Mothur version v.1.30.2 (Schloss et al., 2009). Heatmap was generated using the R package pheatmap.^2^ Phylogenetic trees were constructed using MEGA 7 (Kumar et al., 2016) and visualized using iTOL^3^ (Letunic and Bork, 2016).

### Statistical analysis

Tukey’s test was applied to test differences in the abundance of *dsRed* or *gfp* genes among different treatments. The indices of alpha diversity were shown by the mean ± standard deviation and tested for significant differences between different groups by Duncan’s test. In the beta diversity analysis, the Bray–Curtis dissimilarity matrix was visualized using principal coordinate analysis (PCoA) based on the relative abundance of OTUs in different samples. The line regressions of *gfp* and *dsRed* were performed with the R package ggpmisc.

## Results

### Changes in temperature during composting

Based on the measured temperature profile, both treatments progressed through mesophilic, thermophilic, and maturing phases, as defined in other composting processes (Echeverria et al., 2012; Supplementary Figure S2). The temperatures of the two treatments rose sharply to >55°C on day 2 and maintained the thermophilic phase until day 11, with a maximum temperature of 60.2°C. The temperature of NT treatment decreased from day 12 to room temperature on day 27, indicating that the fermentation process was complete. With an extra heater system, the CT treatment had a thermophilic time of 15 days longer than that of the NT treatment, followed by maturation at room temperature until day 40.

### Fate of donor strain and RP4 plasmid during composting

**Figure 1:**
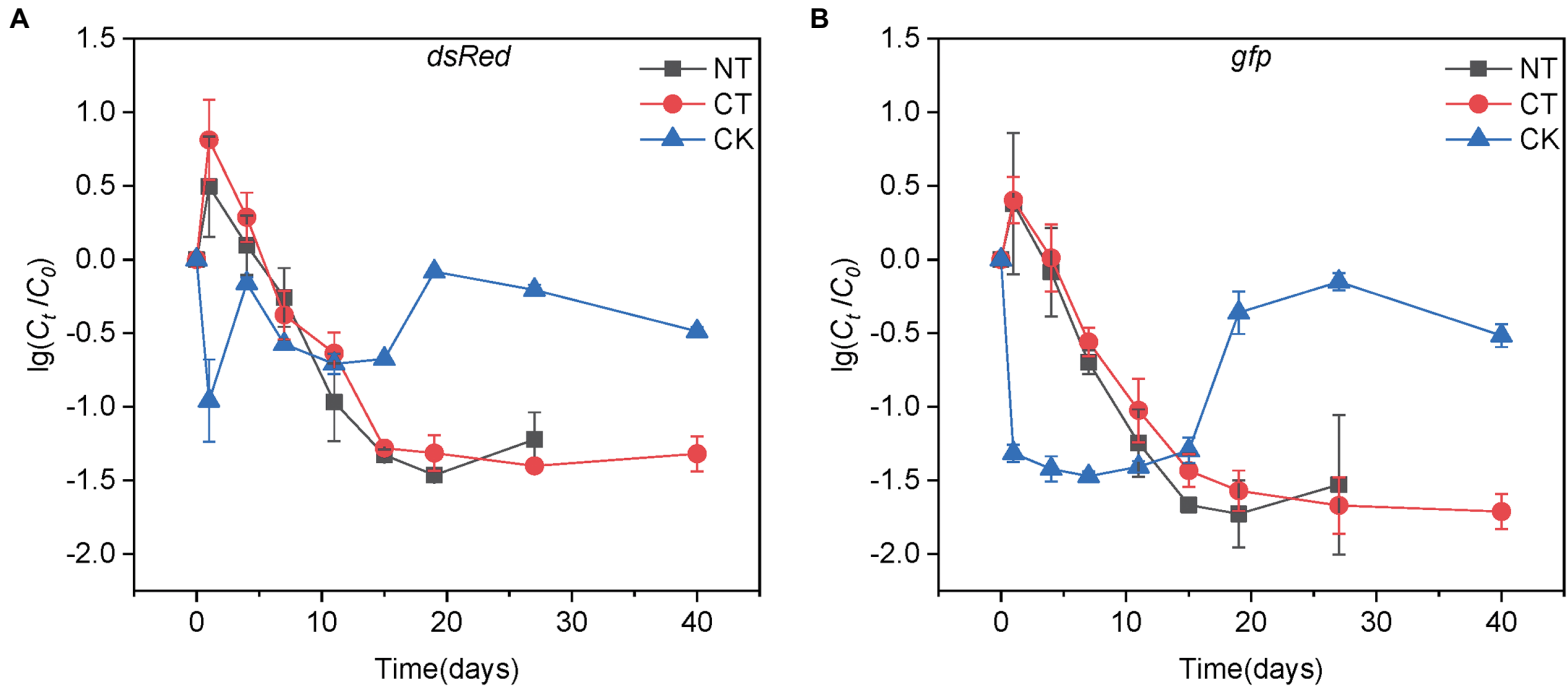
The decay of the donor **(A)** and plasmid **(B)** during normal thermophilic (NT) and continuous thermophilic composting (CT). The log values of the ratios of donor (plasmid) concentration at time *t* (*C_t_*) to the initial concentration (*C_0_*) are shown on the y-axis. The donor and plasmid concentrations are represented by the copy numbers of *dsRed* and *gfp* genes, respectively. Error bars represent the standard deviation (SD) of sampling replicates at each time point.

**Figure 2:**
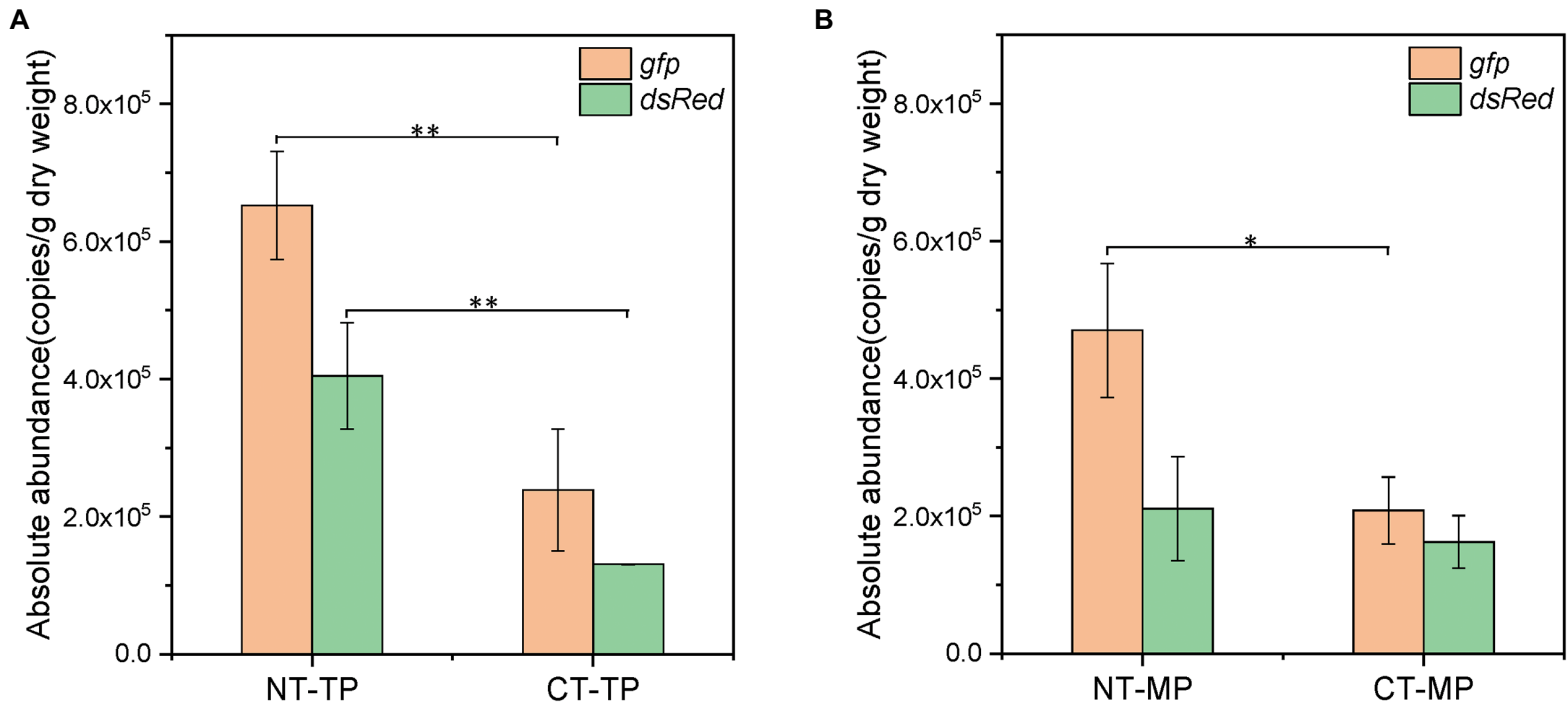
Abundance of *dsRed* and *gfp* genes in two composting treatments at the end of the thermophilic phase **(A)** and mature phase **(B)**. ^*^p < 0.05; ^**^p < 0.01 (Tukey’s test); NT, normal thermophilic composting; CT, continuous thermophilic composting; TP, thermophilic phase; MP, mature phase.

The target genes *dsRed* and *gfp* were quantified by ddPCR to determine the abundance of *P. putida* and RP4 plasmids in the compost samples. As shown in Figure 1, both *P. putida* and plasmid RP4 showed a similar decreasing trend in both the NT and CT treatments. The abundance of *dsRed* and *gfp* in the NT treatment rapidly decreased by 1.46 and 1.73 log to 1.13 × 10^5^ and 2.14 × 10^5^ copies/g DW within 19 days, respectively, and then increased slightly to 2.11 × 10^5^ and 4.70 × 10^5^ copies/g DW on day 27, respectively. In the CT treatment, the *dsRed* abundance decreased by 1.40 log to 1.31 × 10^5^ copies/g DW after 27 days of composting, and increased slightly to 1.63 × 10^5^ copies/g DW on day 40. The abundance of *gfp* continuously decreased by 1.71 log to 2.08 × 10^5^ copies/g DW on day 40. The patterns of *P. putida* and RP4 plasmid decreases in both NT and CT treatments are shown as linear regressions in Supplementary Figure S3. Both *P. putida* and RP4 fates showed biphasic decay behaviors during composting, which was similar to a study in soil by Fan et al. (2019). A linear regression equation was used to calculate the D-values of *dsRed* and *gfp* during composting (Supplementary Table S2). The results showed that the D-values of the target genes in the two treatments in phase I were approximately 7.0 days. After the thermophilic phase, the absolute abundance of *dsRed* and *gfp* in the CT treatment was significantly lower than that in the NT treatment, which was only 32.4% and 36.6% of that in the NT treatment, respectively (Tukey’s test, *p* < 0.01, Figure 2). At the end of the mature phase, the absolute abundance of *dsRed* and *gfp* in the CT treatment was 77.1% and 44.3% of that in the NT treatment, respectively. The results showed that prolonging the thermophilic phase of composting was beneficial to the decay of donor bacteria and RP4 plasmids. After the thermophilic phase, the removal efficiencies of *dsRed* and *gfp* were 96.0% and 97.7% with the CT treatment, and 87.7% and 93.8% with the NT treatment, respectively. At the end of the maturation phase, the removal efficiencies of *dsRed* and *gfp* in the CT treatment (95.1% and 98.0%, respectively) were slightly higher than those in the NT treatment (93.6% and 95.5%, respectively; Supplementary Figure S4). The results showed that thermophilic composting could effectively remove *dsRed* and *gfp*, and that the removal efficiency of CT was greater than that of NT.

Based on the target gene abundance tested using ddPCR, we calculated the SP in the composting samples to estimate the spread and proliferation potential of RP4 plasmid during the composting process. The results showed that the SP in the raw material (IP sample) was 3.91 × 10^−4^ transconjugants per recipient (TC/R), whereas both the NT- and CT-treated samples decreased from 10^−4^ to 10^−6^ (TC/R) after composting. However, the SP in CT-treated samples was 2.54 × 10^−6^ (TC/R) in the final composts, which was lower than that of the NT treatment (Supplementary Table S3). Our results indicated that prolonging the thermophilic time was more favorable for reducing the conjugative transfer potential of plasmid RP4 than normal composting.

### The profile of transconjugants community of RP4 plasmid during composting

**Figure 3:**
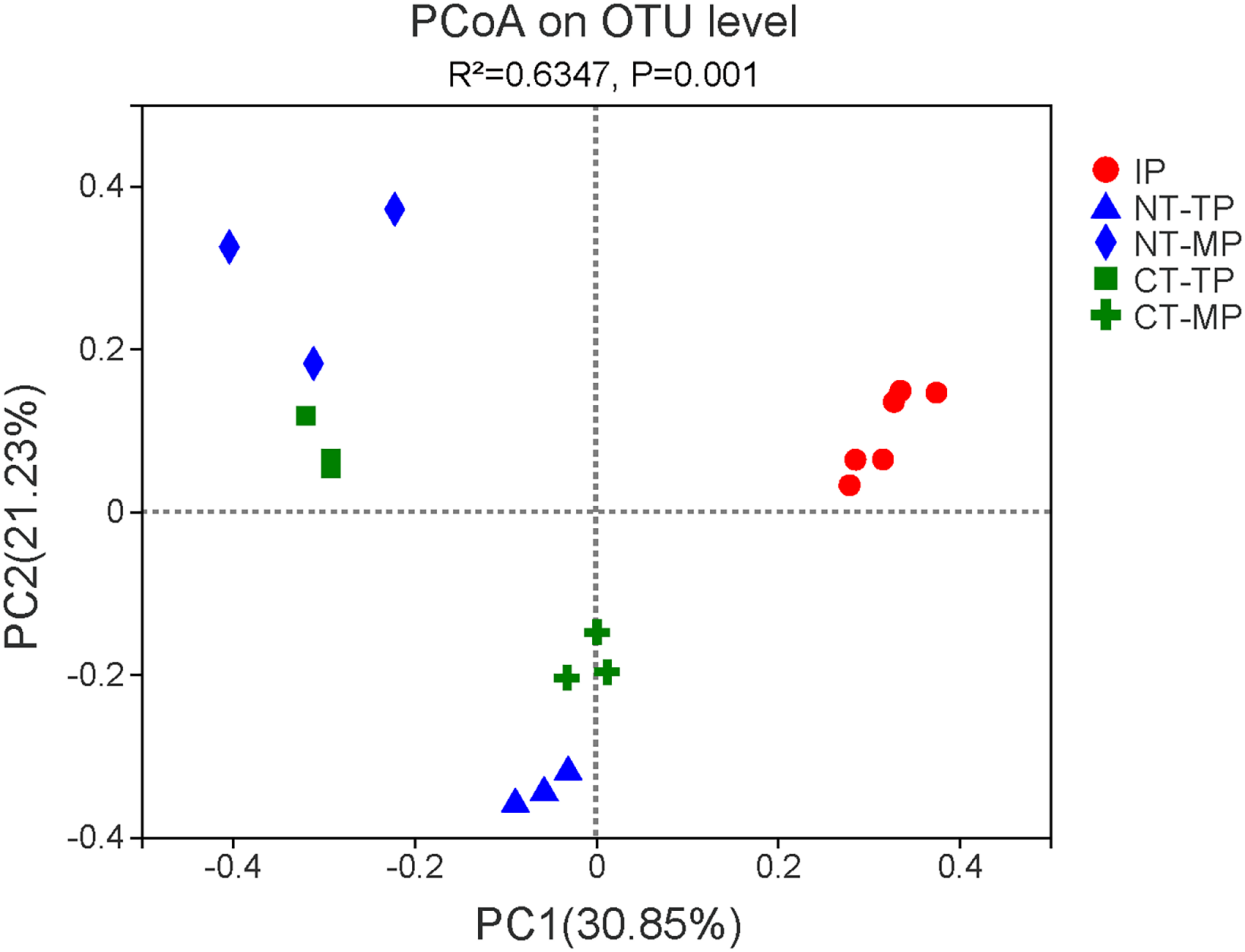
The overall distribution pattern of operational taxonomic unit (OTU)-based transconjugant community dissimilarity in the normal thermophilic (NT) and continuous thermophilic composting (CT). Principal coordinate analysis (PCoA) of beta diversity based on the Bray–Curtis distance analysis of the OTU level. IP, TP, and MP represent the initial, thermophilic, and mature phases, respectively.

Combined with flow cytometry and 16S rRNA gene amplicon sequencing, the community composition of transconjugants obtaining RP4 plasmid was identified. In all 18 samples, 1,093,566 effective sequences were obtained after quality control. The effective sequences were clustered at 97% identity into OTUs, and 220 transconjugant OTUs were identified. Sobs, Chao, Ace, Shannon, Simpson, and Good’s coverage indices were used to assess the alpha diversity (Table 1). In our study, the Good’s coverage was ≥99% for all samples, indicating that the current sequencing depth was sufficient to saturate the transconjugant diversity. Compared with normal composting, prolonging the thermophilic phase of composting significantly decreased the species richness (Sobs, Ace, and Chao indices) and diversity (Shannon index) of RP4 plasmid transconjugants at the end of the thermophilic phase (*p* < 0.05). PCoA based on Bray–Curtis distance at the OTU level showed that the community structure of transconjugants was significantly different among the five groups (Figure 3, Adonis analysis, *p* = 0.001). The taxonomic compositions of the thermophilic (TP samples) and maturation (MP samples) phases were both significantly different from those of the raw material (IP samples), which indicated that composting could shape the structure of RP4 plasmid transconjugants. Because of the prolonged thermophilic phase, the transconjugants of the thermophilic and mature phases between the CT and NT treatments differed from each other.

**Table 1:** Alpha-diversity indexes of bacteria in different composting treatments.

Sample	Sobs	Ace	Chao	Shannon	Simpson	Coverage
IP	79.67 ± 20.58^b^	89.04 ± 11.47^b^	85.64 ± 19.34^b^	3.03 ± 0.47^a^	0.12 ± 0.06^a,b^	0.99 ± 0.00
NT-TP	129.67 ± 3.51^a^	137.17 ± 5.49^a^	136.82 ± 4.92^a^	3.33 ± 0.07^a^	0.07 ± 0.00^b^	0.99 ± 0.00
NT-MP	87.67 ± 11.59^a,b^	89.27 ± 11.84^b^	88.89 ± 11.43^b^	2.24 ± 0.88^b^	0.28 ± 0.26^a^	0.99 ± 0.00
CT-TP	62.33 ± 11.85^b^	65.29 ± 11.43^c^	66.79 ± 16.69^b^	2.29 ± 0.09^b^	0.21 ± 0.02^a,b^	0.99 ± 0.00
CT-MP	67.00 ± 15.62^b^	69.57 ± 17.39^b,c^	68.73 ± 17.10^b^	2.70 ± 0.20^a,b^	0.13 ± 0.03^a,b^	0.99 ± 0.00

Data are presented as mean ± standard deviation (SD). Different lowercase letters indicate a significant difference at *p* < 0.05 level (Duncan’s new multiple range test). NT and CT represent normal and continuous thermophilic composting, respectively; IP, TP, and MP represent the initial, thermophilic, and mature phases, respectively.

**Figure 4:**
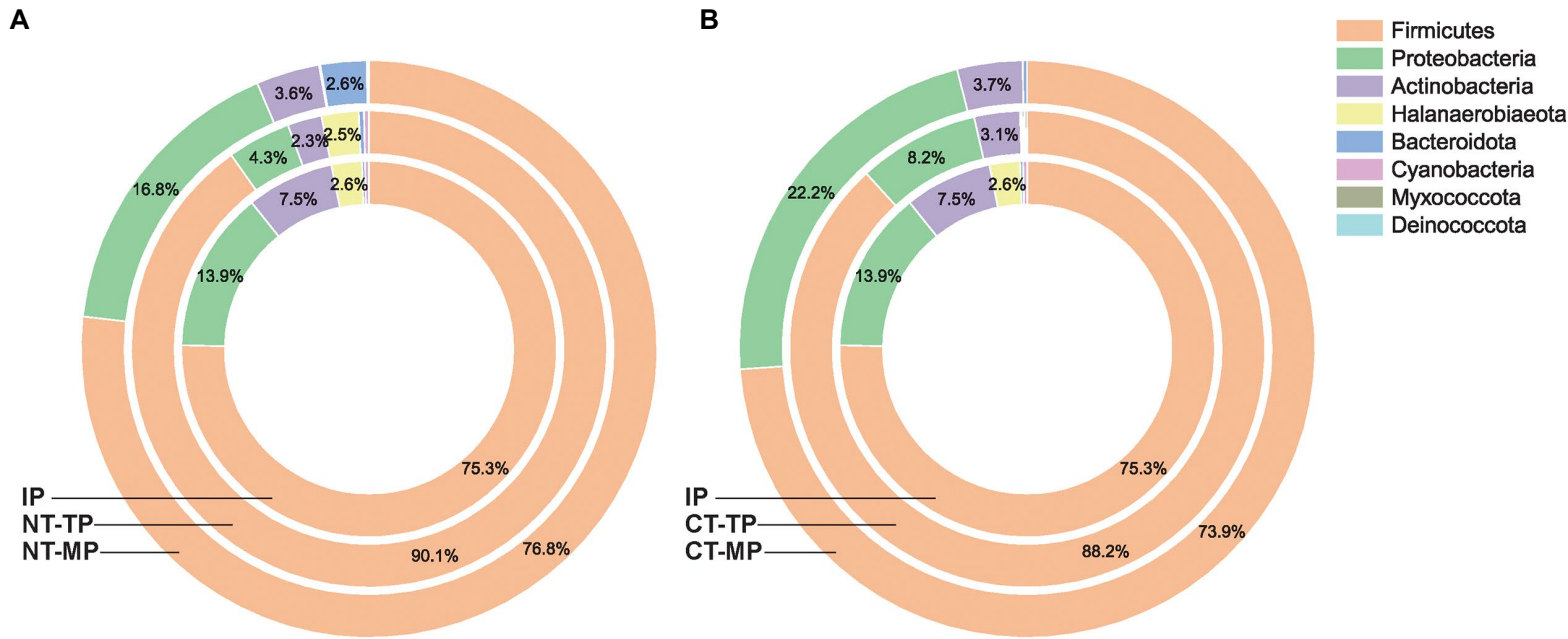
Dynamic changes in community composition of sorted transconjugant pools at the phylum level in the normal thermophilic **(A)** and continuous thermophilic composting **(B)**. NT and CT represent normal and continuous thermophilic composting, respectively; IP, TP, and MP represent the initial, thermophilic, and mature phases, respectively.

Across all samples, 220 permissive OTUs distributed over eight phyla were identified: Firmicutes, Proteobacteria, Actinobacteria, Halanaerobiaeota, Bacteroidota, Cyanobacteria, Myxococcota, and Deinococcota, and thermophilic composting had an obvious influence on the community composition of transconjugants (Figure 4). At the phylum level, the phylogenetic compositions of all the transconjugant pools were similar, but the exact community compositions differed. Firmicutes accounted for 75.3%–90.1% of RP4 plasmid transconjugants, which was the dominant phylum in all samples. High temperature in the thermophilic phase inhibited the growth of transconjugants from Proteobacteria and Actinobacteria, and their relative abundances decreased after the thermophilic phase. However, Proteobacteria transconjugants showed an obvious rebound phenomenon at the mature phase, with the relative abundance increasing from 4.3% to 16.8% in the NT treatment and from 8.2% to 22.2% in the CT treatment. Compared with the NT, the CT treatment had fewer Bacteroidota and Halanaerobiaeota at the end of the mature phase, indicating that CT treatment more efficiently inhibited the growth of Bacteroidota and Halanaerobiaeota, obtaining RP4 plasmid in the final composts. Halanaerobiaeota was completely removed after composting, indicating that prolonged thermophilic time could effectively inhibit Halanaerobiaeota bacteria from obtaining the RP4 plasmid.

**Figure 5:**
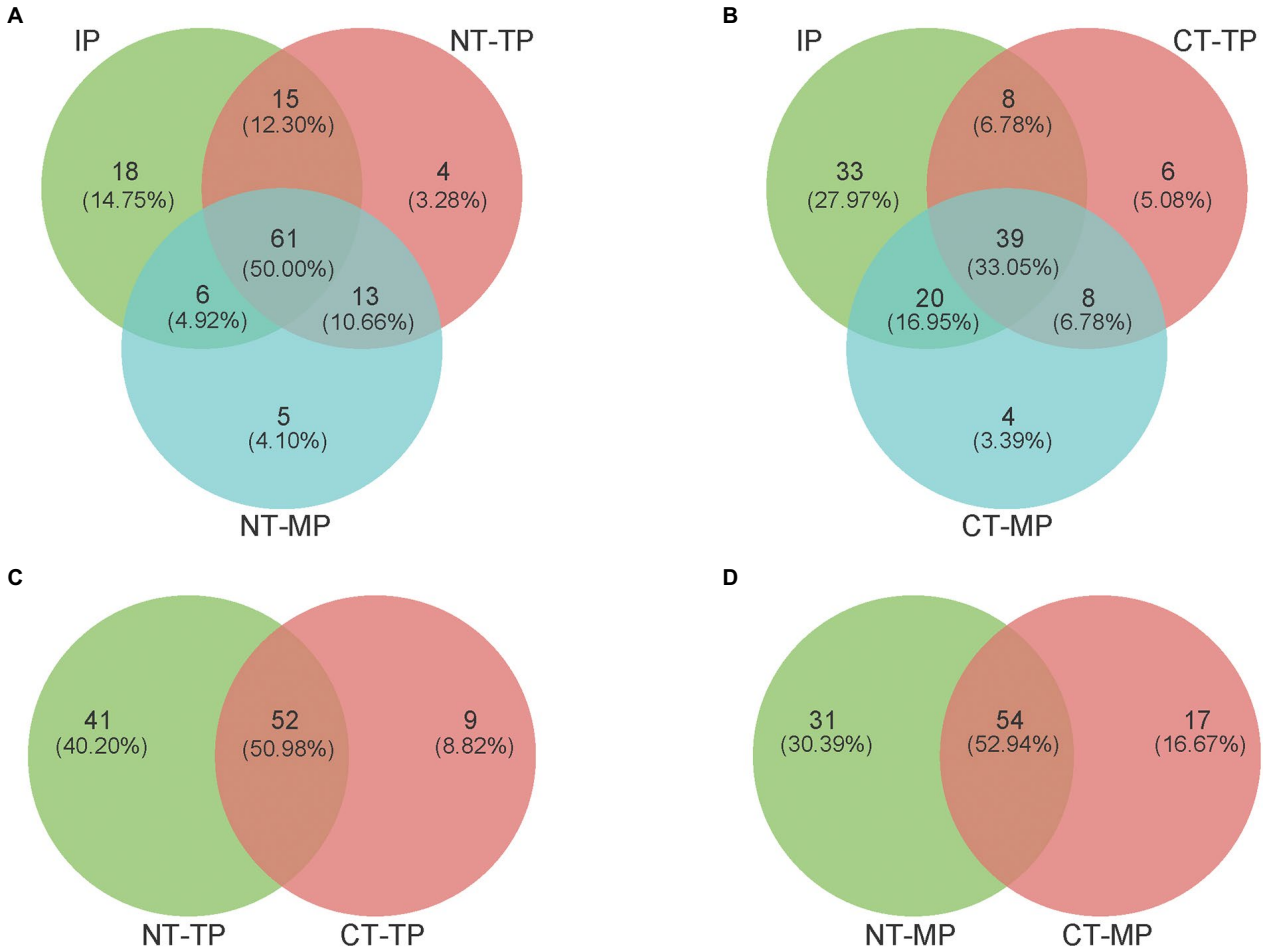
Venn diagrams reveal the shared and unique numbers of transconjugant pools for plasmid RP4 transferred at the genus level. **(A,B)** Show the number of shared and unique genera among the three composting phases (IP, TP, and MP) in the NT and CT, respectively. **(C,D)** Diagrams show the number of shared and unique genera between NT and CT at the end of the thermophilic and mature phase, respectively. NT and CT represent normal and continuous thermophilic composting, respectively; IP, TP, and MP represent the initial, thermophilic, and mature phases, respectively.

For the number of genera in the composts, the transconjugant pools of the two composting treatments (NT and CT) shared 50.00% and 33.05% genera among the three composting phases (IP, TP, and MP), respectively (Figures 5A,B). Shared genera during composting could be considered the core host community of the RP4 plasmid. Fewer shared genera represented a smaller core transfer host range in the CT treatment than in the NT treatment. For the unique genera in composts, at the end of the mature phase, both NT and CT had notably fewer unique host genera (five and four genera, respectively) than that (46 genera) in CK samples without composting (Supplementary Figure S5). Unique genera represent possible new recipients for the RP4 plasmid, so fewer unique genera at the end of composting indicated that composting could efficiently control the conjugative transfer opportunity to new genera in organic fertilizer. However, at the end of the thermophilic phase of composting, the NT and CT treatments shared 52 genera, with 41 unique genera to NT and only 9 unique genera to CT (Figure 5C); at the end of the mature phase, the NT and CT treatments shared 54 genera, with 31 unique genera to NT and 17 unique genera to CT (Figure 5D), indicating significantly decreased diversity of transconjugants in the CT treatment. These results show that CT treatment is more conducive to reducing the diversity of transconjugants than NT.

**Figure 6:**
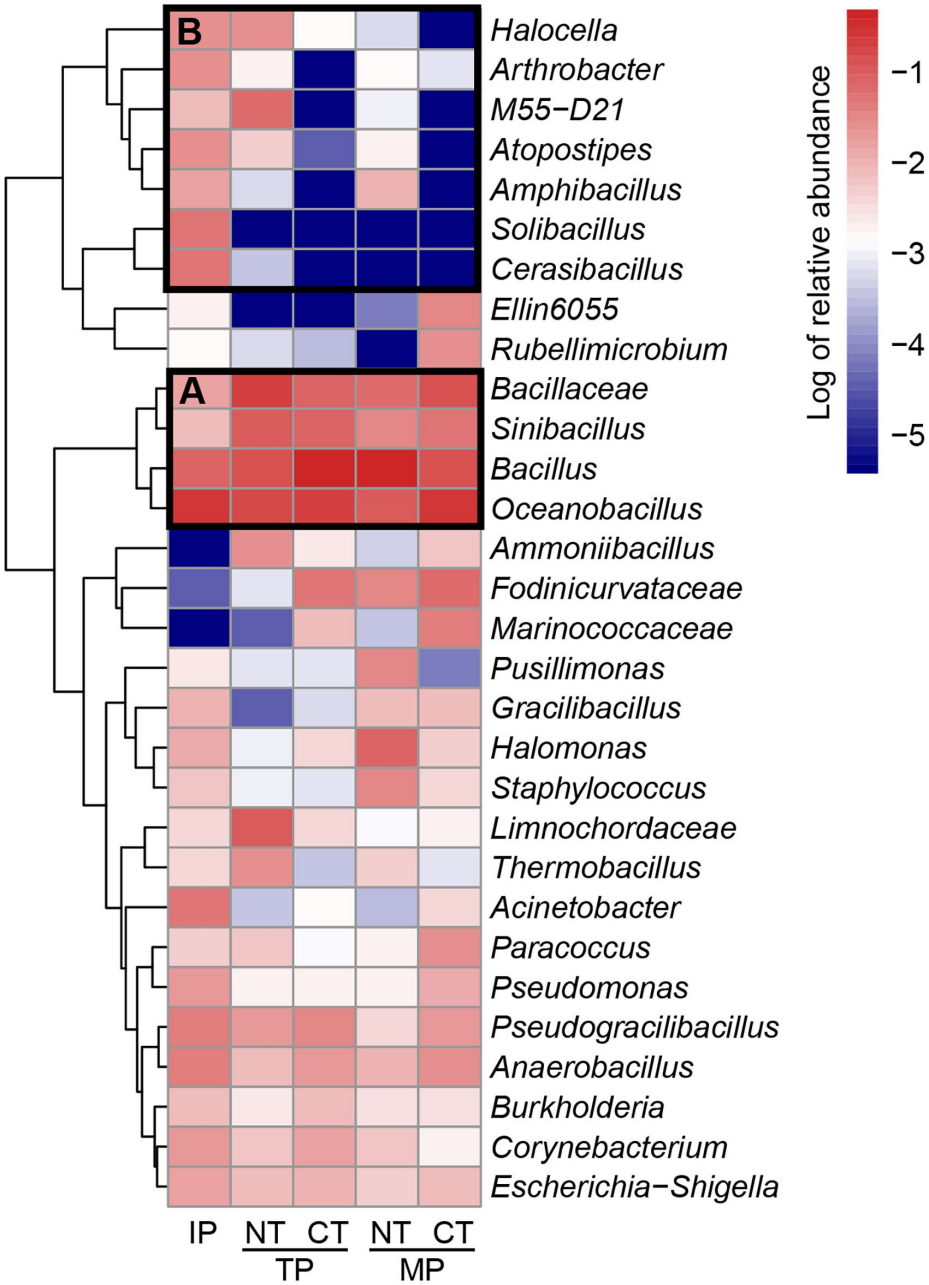
Relative abundance of top 30 abundant genera in the transconjugant pools during normal thermophilic (NT) and continuous thermophilic composting (CT). IP, TP, and MP represent the initial, thermophilic, and mature phases, respectively. **(A)** dominant conjungants in all samples; **(B)** removal of RP4 plasmid hosts.

The heatmap of the top 30 genera was generated to visualize the transconjugant community composition better at the genus level (Figure 6). The genera in group A, such as *Oceanobacillus*, *Bacillus*, *Bacillaceae*, and *Sinibacillus*, were the dominant conjugants in all the samples during composting. *Oceanobacillus*, accounting for 27.41% of the total relative abundance of transconjugants, was the most abundant host in the raw material. *Bacillus* (44.66%) and *Bacillaceae* (19.66%) became the most abundant hosts in the thermophilic phase in CT and NT, respectively. After the maturation phase, *Bacillus* (48.41%) became the most dominant genus in the NT treatment, followed by *Oceanobacillus* (9.66%), *Halomonas* (7.65%), *Bacillaceae* (5.92%), *Fodinicurvataceae* (3.41%), *Sinibacillus* (3.07%), and *Staphylococcus* (3.07%). *Oceanobacillus* (30.23%) was still the most dominant genus in the CT treatment, followed by *Bacillaceae* (13.63%), *Bacillus* (12.65%), *Fodinicurvataceae* (6.37%), *Sinibacillus* (5.49%), *Marinococcaceae* (3.72%), and *Paracoccus* (2.85%). The genera in group B represented the removal of RP4 plasmid hosts such as *Halocella*, *Solibacillus*, *Atopostipes*, *Amphibacillus*, and *Cerasibacillus* during composting. In particular, some opportunistic pathogenic bacteria such as *Corynebacterium*, *Escherichia, Shigella*, *Staphylococcus*, *Pseudomonas*, and *Burkholderia* received the ARG-carrying RP4 plasmid and were partially removed after composting.

**Figure 7:**
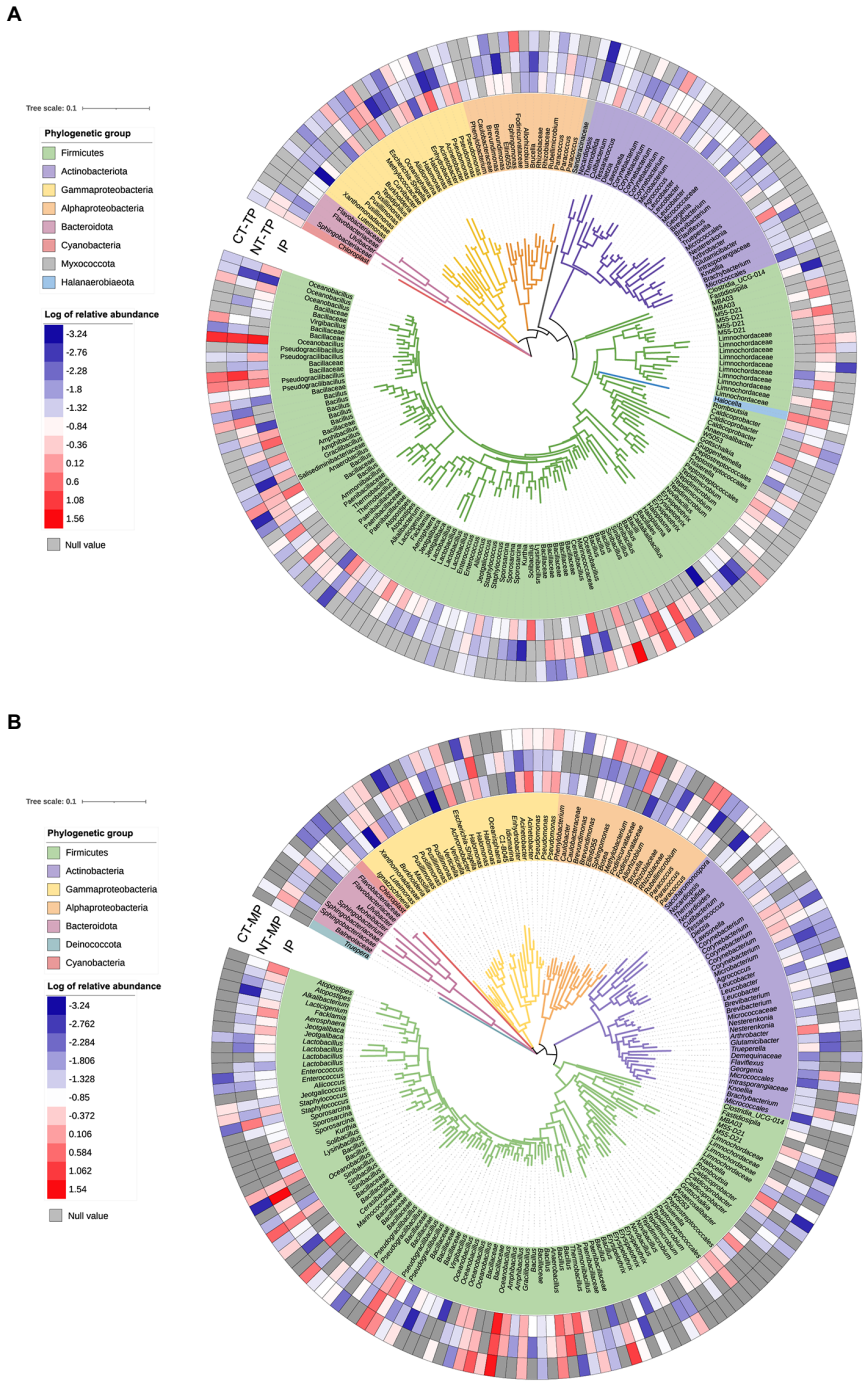
Phylogenetic tree showing the identified transconjugant OTUs that contributed more than 0.01% in normal thermophilic (NT) and continuous thermophilic composting (CT). **(A)** Showing transconjugants in the thermophilic phase. **(B)** Showing transconjugants in the mature phase. Colors of the branches mark different phylogenetic groups. Heatmap circles at the periphery of the tree represent the log of relative abundance of the OTU in the transconjugant pools. IP, TP, and MP represent the initial, thermophilic, and mature phases, respectively.

Phylogenetic trees were constructed from OTUs with greater than 0.01% relative abundance in the transconjugant pools. As shown in Figure 7, Firmicutes comprised the major OTUs, accounting for more than 70% of the bacterial community structure. The RP4 plasmid was transferred from donor strain to a wide range of recipients, including the phylogenetically distant species. Notably, some dominant Gram-positive transconjugants were also identified, such as Firmicutes and Actinobacteria (Figure 7).

## Discussion

### Continuous thermophilic composting is more effective at removing donor strain and plasmid RP4 compared with normal thermophilic composting

Composting is an effective way to utilize waste resources, and high temperatures are an important factor for the inactivation of pathogenic microorganisms in organic waste (Nakasaki et al., 1985). In our study, *P. putida* carrying the RP4 plasmid was used as a model strain to study the dynamic changes of donor and broad-host-range plasmids in the compost microcosm under a prolonged thermophilic phase. By quantifying the marker genes *dsRed* and *gfp* using ddPCR, we observed that *P. putida* and RP4 plasmids in CT demonstrated a biphasic pattern of decay, which included a fast decay during the first 15 days followed by a slow decay after the 15th day (Supplementary Figure S3). *Pseudomonas putida* and RP4 plasmids in the NT treatment had a similar first decay to the CT treatment, but instead of decay, they both increased at the second stage without prolonging the thermophilic phase (Supplementary Figure S3). These results suggest that thermophilic composting could effectively remove donor bacteria and RP4 plasmids by preventing their rebound during the mature phase. Linear regression equation was used to calculate the D-values of *P. putida* and RP4 plasmids for all treatments (Supplementary Figure S3; Supplementary Table S2). During composting, *P. putida* had a larger D-value (6.9–7.4 days) than *E. coli* (0.27–4.82 days; Thomas et al., 2020). *Pseudomonas* is a Gram-negative mesophilic bacterium that is sensitive to high temperatures during composting. However, *Pseudomonas*, including the pathogen *Pseudomonas aeruginosa*, could still be isolated from mature composts because of the temperature difference between the inner and outer areas of composting (Kaszab et al., 2011). However, the abundance of *gfp* rebounded at the maturation stage in the NT treatment but not in the CT treatment, which may be caused by the regrowth of some RP4 plasmid hosts in the NT treatment. The result was consistent with the fact that the richness of RP4 plasmid transconjugants in the CT treatment was lower than that in the NT treatment during the maturation phase (Table 1). The better removal effect of CT on ARG-carrying RP4 plasmid may be due to the elimination of some non-thermophilic ARG hosts or inhibition of HGT by high temperatures (Miller et al., 2016; Qian et al., 2016).

In addition to reducing the abundance of ARB and their plasmids, the high composting temperature mitigated the frequency of conjugation. SP (3.91 × 10^−4^ TC/R) in the raw material is similar to the study (4.65 × 10^−4^ TC/R) of Fan et al. (2019); however, in contrast to the final increase in the soil environment, SP significantly decreased to 2.54–6.73 × 10^−6^ (TC/R) at the end of composting (Fan et al., 2019). Compared with mesophilic digestion, thermophilic digestion is more conducive to resisting the HGT of ARGs in bacterial communities (Miller et al., 2016). Although our results directly demonstrate that composting can effectively control the transfer rate of broad-host conjugative plasmids, some studies indicate that composting is not effective for all conjugative plasmids (Le Devendec et al., 2016), particularly *sul1*-related plasmids that may survive transfer to heat-resistant bacteria (Lin et al., 2021).

The initial abundance of *dsRed* and *gfp* were averaged at 3.30 × 10^6^ and 1.04 × 10^7^ copies/g DW, respectively, and increased slightly on the first day after fermentation. This could be due to the division of the donor strain, consistent with previous studies (Henschke and Schmidt, 1990; Fan et al., 2019). We found that plasmid transfer occurred at the beginning of composting using flow cytometric analysis and sorting of transconjugants from the first day samples. A previous study reported that transconjugants appeared immediately after *P. putida* BH(RP4) or *E. coli* C600(RP4) was introduced into soil microcosms (Inoue et al., 2009). The ddPCR results of *dsRed* and *gfp* show that the removal efficiencies of donor strains and RP4 plasmid in CT were higher than that in NT composting. The persistence of ARG-carrying plasmids during manure storage and composting were observed, and a high temperature of composting could inhibit the transfer of RP4 plasmid based on culture-dependent methods (Guan et al., 2007; Le Devendec et al., 2016). Our culture-independent results add more evidence that composting could reduce the abundance and transfer of ARG-carrying plasmids through prolonging the thermophilic phase. Besides the quantities of donors with plasmids, the composition of the recipient pool is another important factor affecting plasmid HGT (Lopatkin et al., 2016). It has been reported that the HGT frequency is usually affected by cell density (Johnsen and Kroer, 2007), therefore HGT may vary in different composting phases because of a different recipient community.

### Shift of transconjugant community during composting and prolonging thermophilic phase influences

The broad-host-range plasmid RP4 has an extremely broad transfer range, including both Gram-negative and Gram-positive bacteria (Musovic et al., 2014; Klümper et al., 2015; Li et al., 2018). Here, we demonstrated that plasmid RP4 transfers to a diverse fraction of the bacterial community during composting. PCoA analysis showed that the composition of the transconjugant community differed between the NT and CT treatments, and changed with different fermentation periods (Figure 3). Compared with NT, prolonging the thermophilic phase leads to a decrease in the diversity and richness of transconjugant communities (Table 1). The accumulated temperature, including the temperature intensity and duration of the thermophilic phase, could profoundly affect the bacterial community (Liu et al., 2021). Liao et al. (2018) found that a higher composting temperature had better efficiency in reducing the abundance of potential ARG bacterial hosts such as *Bacillus* which was also enhanced in our composting especially at the thermophilic phase (Figure 6). Continuous thermophilic composting or external heating had a better performance at reducing human pathogenic bacteria (Qian et al., 2016; Keenum et al., 2021). Both a high temperature and prolonged thermophilic time may supply a small size recipient pool for the conjugative plasmid, so the total number and abundance of transconjugant genera carrying plasmid RP4 in the CT treatment were significantly lower than those in the NT treatment at the end of both the thermophilic and maturation phases (Figures 5C,D, 7). This finding clearly revealed that the transfer of multidrug-resistant plasmids could effectively be weakened by prolonging the thermophilic period.

Phylogenetic analysis of transconjugants sorted by flow cytometry indicated that RP4 disseminated to a broad transfer range (covering eight phyla) of hosts during composting, including the phylogenetically distant group (Figure 7). Firmicutes was the most abundant phylum, which differs from previous studies reporting that Proteobacteria is the predominant host for the transfer of RP4 plasmid in soil (Klümper et al., 2015, 2017; Fan et al., 2019), plant rhizosphere (Xu et al., 2021), and wastewater treatment plants (Li et al., 2018). On the other hand, it is consistent with the results of major hosts at mesophilic and thermophilic phases from a transcriptional resistome study of manure composting (Wang et al., 2017). These results revealed that environmental niches and experimental conditions could determine the host profile of multidrug-resistant conjugative plasmids. Firmicutes was significantly enriched during the thermophilic phase of both composting treatments. The specific property of Firmicutes to form endospores may be an explanation for this enhancement (de Hoon et al., 2010); therefore, they are tolerant to high temperature (Hartmann et al., 2014) and can survive environmental stresses (Barnard et al., 2013), which is the main reason why Firmicutes is the dominant RP4 plasmid-carrying bacteria during composting. Based on co-occurrence network analysis and conjugative experiments, Firmicutes, such as *Lactobacillus*, *Bacillus,* and *Staphylococcus*, are also potential ARG hosts after composting (Lin et al., 2021; Sardar et al., 2021; Zhou et al., 2021). Proteobacteria was the second predominant phylum of transconjugants in our samples, and its relative abundance, including Alphaproteobacteria and Gammaproteobacteria, increased after the mature phase. Alphaproteobacteria, including *Fodinicurvataceae*, *Paracoccus*, *Sphingomonadaceae* (*Ellin6055*), and *Rubellimicrobium*, made major contributions to the “rebound” of Proteobacteria at the end of composting (Figure 7B). *Fodinicurvataceae* and *Rubellimicrobium* are thermophilic bacteria associated with humic acid conversion during composting (Ye et al., 2021; Fu et al., 2022). *Sphingomonadaceae (Ellin6055)* and *Paracoccus* are two abundant hosts of another broad-host-range plasmid (pKJK5) in the soil (Klümper et al., 2017). Notably, we found transfer from the used Gram-negative donor strain (*Pseudomonas*) to a wide variety of Gram-positive bacteria belonging to Firmicutes and Actinobacteria (Figure 7), indicating the inter-Gram gene transfer during composting. The most dominant family containing Gram-positive bacteria was Bacillaceae, including *Bacillus*, *Oceanobacillus*, *Sinibacillus*, *Pseudogracilibacillus*, *Anaerobacillus*, and *Cerasibacillus*. Similar RP4 plasmid transfers have been found in the soil and plant endosphere (Klümper et al., 2015; Xu et al., 2021). The shared transfer host bacteria of plasmids among composts, soil, and the plant endosphere indicate a potential risk for plasmid-mediated ARG exchange through fertilizer utilization on farms.

## Conclusion

In this study, we found a biphasic decay behavior of *P. putida* and RP4 plasmid during composting, and prolonging thermophilic composting supplied a slow decay after 15 days. Composting could mitigate the spread of the RP4 plasmid, and the SP_ddPCR_ significantly decreased at the final compost. Prolonging the thermophilic phase could effectively reduce the diversity and richness of the RP4 host community. Different from previously studied niches such as soil, plant rhizosphere, and wastewater, Firmicutes was the predominant host in the composting transconjugant pools. Our findings provide a feasible solution for mitigating the HGT of ARGs during composting and inhibiting the spread of ARGs from animals to farmland.

## Data availability statement

The datasets presented in this study can be found in online repositories. The names of the repository/repositories and accession number(s) can be found at: https://ngdc.cncb.ac.cn/gsa/, CRA006962.

## Author contributions

XW and GZ conceived the idea, edited the manuscript, and made substantial contribution to the conception of the work. LS, TQ, and HG performed the composting, ddPCR, and FACS. LS, TQ, and YG performed sequencing and its analysis. MG performed the statistical analysis. LS and TQ wrote the original manuscript. All authors contributed to the article and approved the submitted version.

## Funding

This work was supported by Beijing Natural Science Foundation (grant number 6222013), the Special Program for Creative Ability Foundation of BAAFS (grant number KJCX20210424), and Beijing Agriculture Innovation Consortium (BJJQ-G08).

## Conflict of interest

The authors declare that the work was conducted in the absence of any commercial or financial relationships that could be construed as a potential conflict of interest.

## Publisher’s note

All claims expressed in this article are solely those of the authors and do not necessarily represent those of their affiliated organizations, or those of the publisher, the editors and the reviewers. Any product that may be evaluated in this article, or claim that may be made by its manufacturer, is not guaranteed or endorsed by the publisher.

## Footnotes

^1^ http://rdp.cme.msu.edu/

^2^ http://www.r-project.org/

^3^ http://itol.embl.de/
