# Supplementary material for: Enhancing control of multidrug-resistant plasmid and its host community with a prolonged thermophilic phase during composting
Source: Front Microbiol. 2022 Aug 17;13:989085. doi: 10.3389/fmicb.2022.989085 (PMC9428157; doi:10.3389/fmicb.2022.989085)
Supplement: Supplementary file 1 [file Data_Sheet_1.DOCX]

Supplementary Material

**Supplementary Figures:**

**
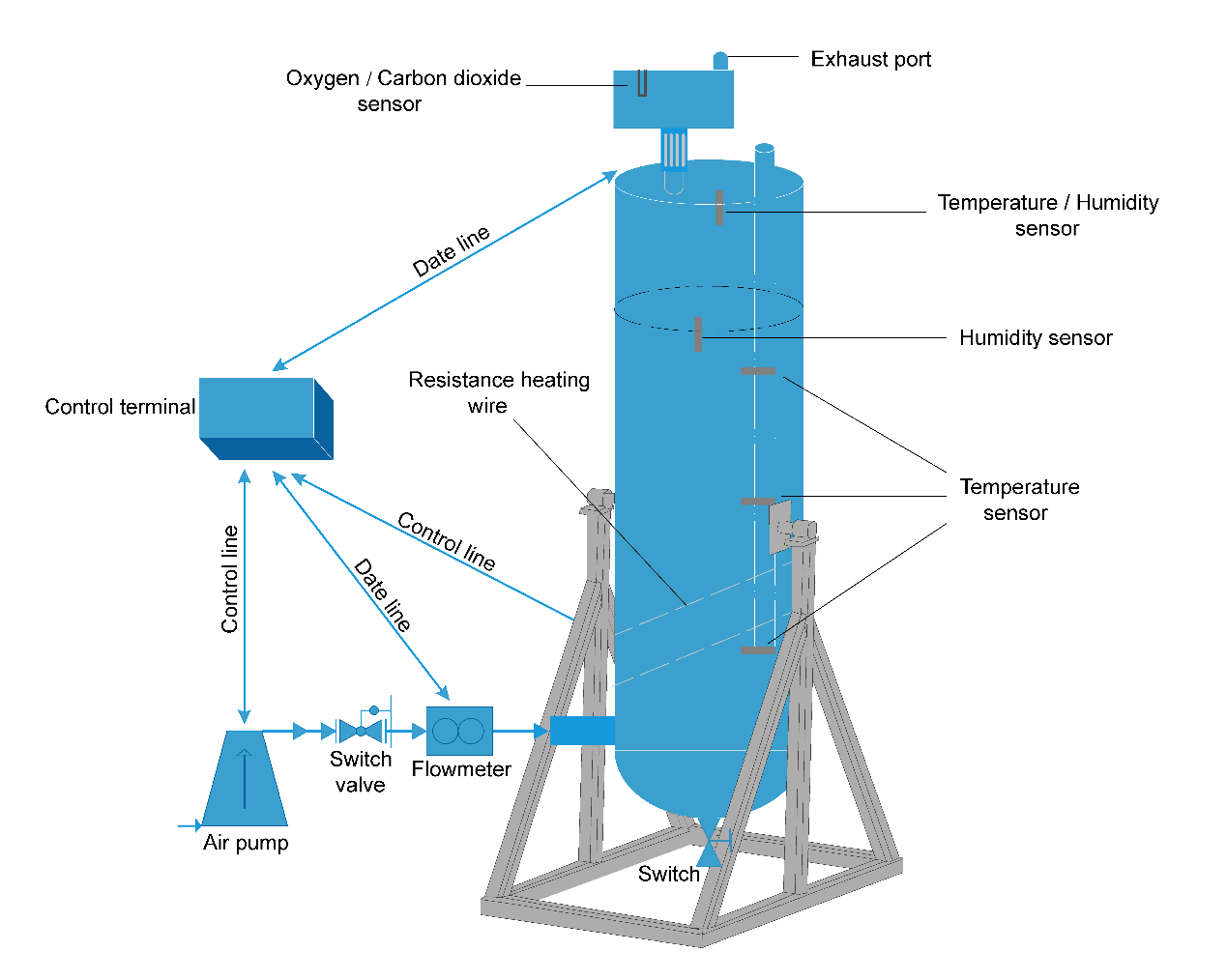
**

**Supplementary Figure S1.** Schematic diagram of the compost reactor.

**Supplementary Figure S2.** Temperature change in the different treatments during normal thermophilic (NT) and continuous thermophilic composting (CT).


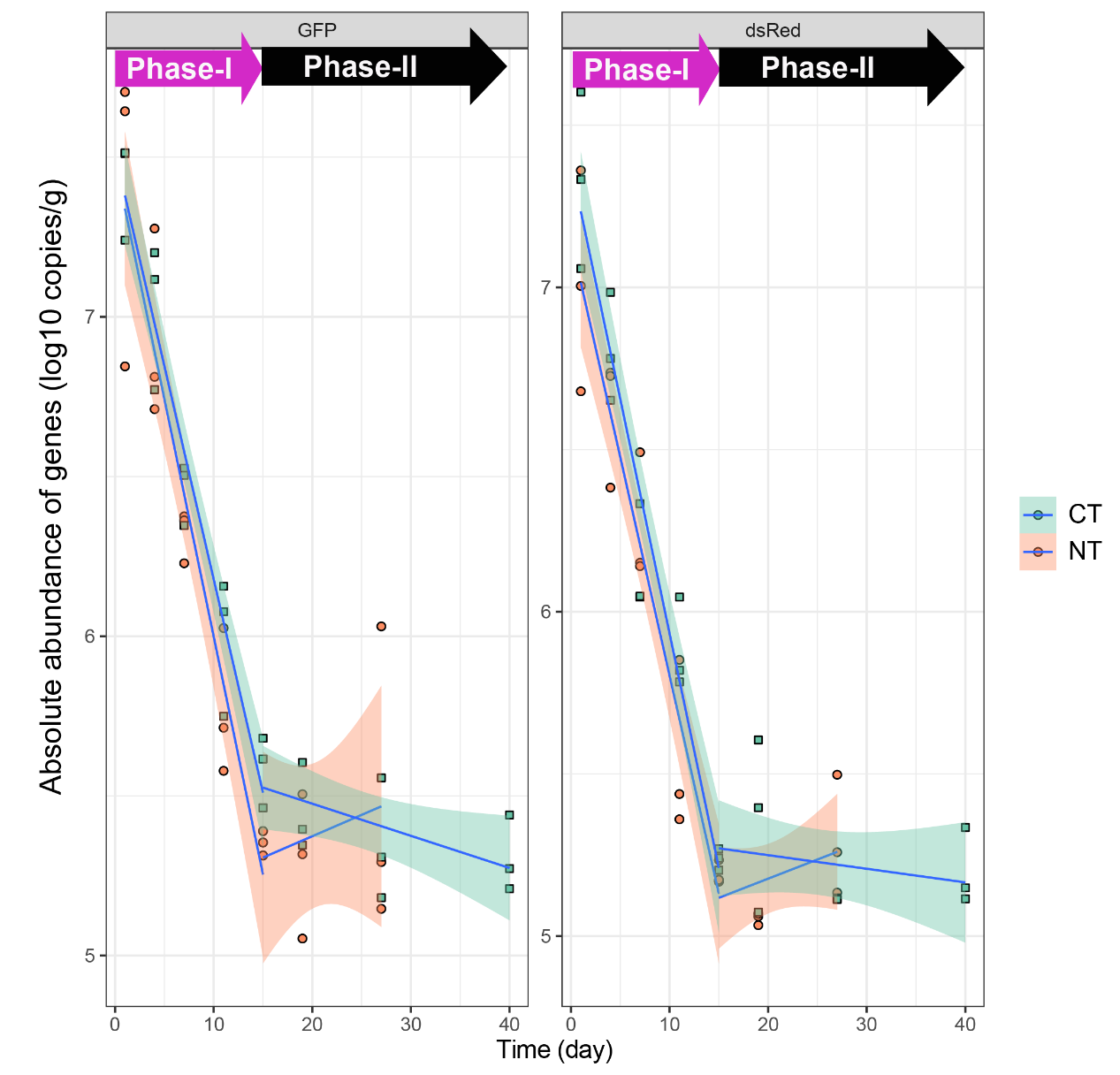


**Supplementary Figure S3.** Decrease in *gfp* and *dsRed* genes during composing with different duration of thermophilic phases.


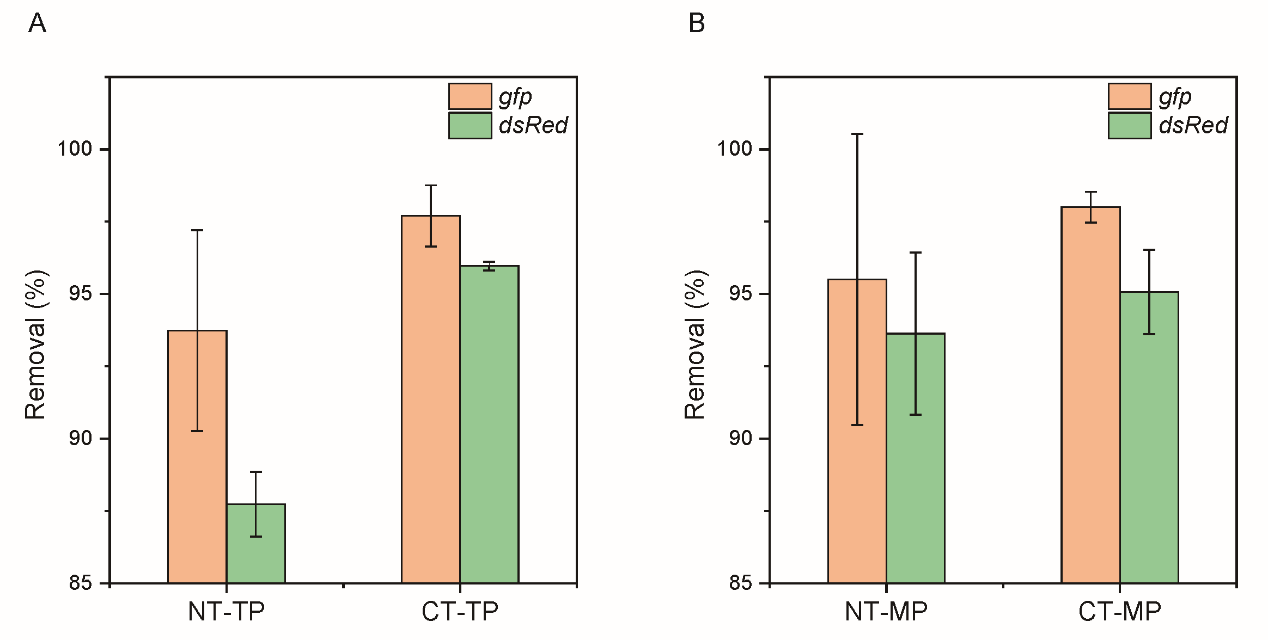


**Supplementary Figure S4.** The removal of *dsRed* and *gfp* genes in two composting treatments at the end of thermophilic phase (**A**) and maturing phase (**B**). Abbreviations: NT and CT represented normal thermophilic and continuous thermophilic composting, respectively.


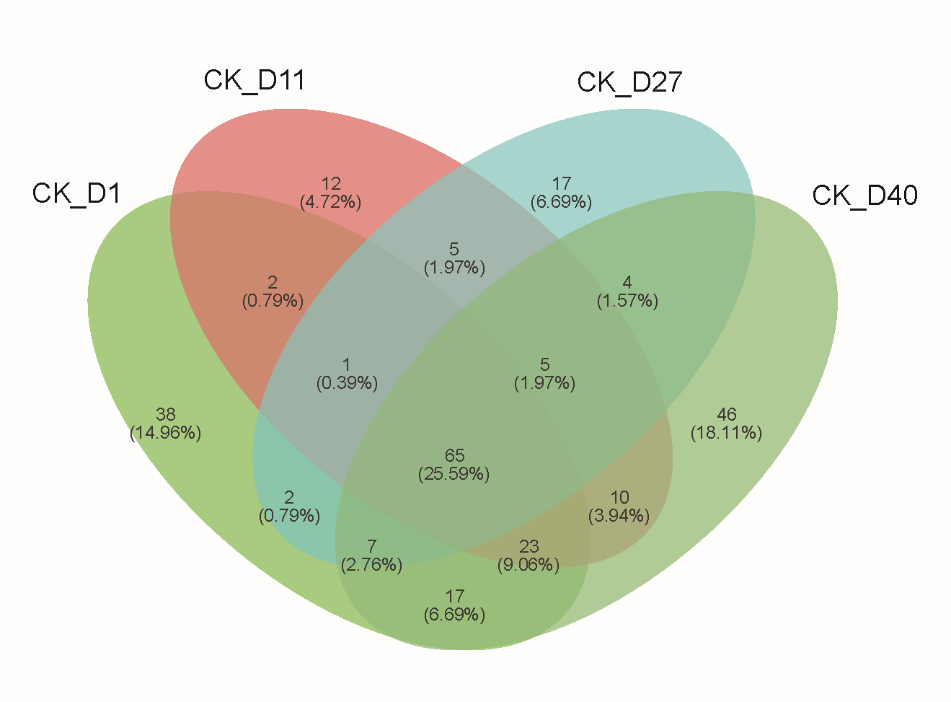


**Supplementary Figure S5.** Venn diagrams at the genus level of the transconjugant pools for plasmid RP4 transferred in CK treatment.

**Supplementary Tables:**

**Supplementary Table S1.** Primer list for ddPCR assay.

| **Gene** | **Sequences 5′ → 3′** | **Tm (°C)** | **References** |
| --- | --- | --- | --- |
| *gfp* | Fw 5′-GAAGATGGAAGCGTTCAA-3′ | 58 | Hale et al., 2015 |
|  | Rv 5′-AGGTAATGGTTGTCTGGTA-3′ |  |  |
| *dsRed* | Fw 5′-TCCTCGTGATGGCGTGTTGA-3′ | 57 | This work |
|  | Rv 5′-AGCTGCACAGGCTTCTTTGC-3′ |  |  |
| 16S rRNA | Fw 5′-GGGTTGCGCTCGTTGC-3′ | 60 | Zhu et al., 2017 |
|  | Rv 5′-ATGGYTGTCGTCAGCTCGTG-3′ |  |  |

**References**

Hale, L., Luth, M., and Crowley, D. (2015). Biochar characteristics relate to its utility as an alternative soil inoculum carrier to peat and vermiculite. *Soil Biol. Biochem*. 81, 228–235. doi: 10.1016/j.soilbio.2014.11.023

Zhu, Y. G., Zhao, Y., Li, B., Huang, C. L., Zhang, S. Y., Yu, S., et al. (2017). Continental-scale pollution of estuaries with antibiotic resistance genes. *Nat. Microbiol*. 2:16270. doi: 10.1038/nmicrobiol.2016.270

**Supplementary Table S2.** Decimal reduction times (D-values) for the *gfp* and *dsRed* genes druing composting.

| **Treatment** | **Gene** | **Decay phase** | **Regression equation^a^** | **D-value**  **(days)** | **R^2^** | ***p*** |
| --- | --- | --- | --- | --- | --- | --- |
| CT | *gfp* | Ⅰ | Y = 7.51－0.134X | 7.46 | 0.943 | <0.001 |
| CT | *gfp* | Ⅱ | Y = 5.68－0.0101X | 100 | 0.303 | 0.037 |
| NT | *gfp* | Ⅰ | Y = 7.49－0.149X | 6.71 | 0.896 | <0.001 |
| NT | *gfp* | Ⅱ | Y = 5.11＋0.0134X | --^#^ | -0.069 | 0.509 |
| CT | *dsRed* | Ⅰ | Y = 5.33－0.0042X | 6.90 | 0.933 | <0.001 |
| CT | *dsRed* | Ⅱ | Y = 7.38－0.145X | 238.09 | -0.02 | 0.397 |
| NT | *dsRed* | Ⅰ | Y = 7.15－0.135X | 7.41 | 0.912 | <0.001 |
| NT | *dsRed* | Ⅱ | Y = 4.94＋0.0118X | --^#^ | 0.08 | 0.234 |

^a^ Y is the absolute abundance of genes (log_10_ copies/g), and X is the time in days.

^#^ The slope of equation was positive number.

Abbreviations: NT and CT represent normal thermophilic and continuous thermophilic composting, respectively.

**Supplementary Table S3.** Decimal reduction times (D-values) for the *gfp* and *dsRed* genes druing composting.

| **Sample** | IP | NT-TP | NT-MP | CT-TP | CT-MP |
| --- | --- | --- | --- | --- | --- |
| SP (TC/R) | 3.91×10^-4^ | 4.71×10^-6^ | 6.73×10^-6^ | 4.25×10^-6^ | 2.54×10^-6^ |

Abbreviations: NT and CT represent normal thermophilic and continuous thermophilic composting, respectively; IP, TP, and MP represent the initial, thermophilic and mature phase, respectively.
